# Selective oxidative stress induces dual damage to telomeres and mitochondria in human T cells

**DOI:** 10.1111/acel.13513

**Published:** 2021-11-09

**Authors:** Ling Wang, Zeyuan Lu, Juan Zhao, Madison Schank, Dechao Cao, Xindi Dang, Lam Nhat Nguyen, Lam Ngoc Thao Nguyen, Sushant Khanal, Jinyu Zhang, Xiao Y. Wu, Mohamed El Gazzar, Shunbin Ning, Jonathan P. Moorman, Zhi Q. Yao

**Affiliations:** ^1^ Center of Excellence in Inflammation, Infectious Disease and Immunity Quillen College of Medicine East Tennessee State University Johnson City Tennessee USA; ^2^ Division of Infectious, Inflammatory and Immunologic Diseases Department of Internal Medicine Quillen College of Medicine East Tennessee State University Johnson City Tennessee USA; ^3^ Hepatitis (HCV/HBV/HIV) Program Department of Veterans Affairs James H. Quillen VA Medical Center Johnson City Tennessee USA

**Keywords:** DNA damage and repair, mitochondria, oxidative stress, T cell senescence, telomeres

## Abstract

Oxidative stress caused by excess reactive oxygen species (ROS) accelerates telomere erosion and mitochondrial injury, leading to impaired cellular functions and cell death. Whether oxidative stress‐mediated telomere erosion induces mitochondrial injury, or vice versa, in human T cells—the major effectors of host adaptive immunity against infection and malignancy—is poorly understood due to the pleiotropic effects of ROS. Here we employed a novel chemoptogenetic tool that selectively produces a single oxygen (^1^O_2_) only at telomeres or mitochondria in Jurkat T cells. We found that targeted ^1^O_2_ production at telomeres triggered not only telomeric DNA damage but also mitochondrial dysfunction, resulting in T cell apoptotic death. Conversely, targeted ^1^O_2_ formation at mitochondria induced not only mitochondrial injury but also telomeric DNA damage, leading to cellular crisis and apoptosis. Targeted oxidative stress at either telomeres or mitochondria increased ROS production, whereas blocking ROS formation during oxidative stress reversed the telomeric injury, mitochondrial dysfunction, and cellular apoptosis. Notably, the X‐ray repair cross‐complementing protein 1 (XRCC1) in the base excision repair (BER) pathway and multiple mitochondrial proteins in other cellular pathways were dysregulated by the targeted oxidative stress. By confining singlet ^1^O_2_ formation to a single organelle, this study suggests that oxidative stress induces dual injury in T cells via crosstalk between telomeres and mitochondria. Further identification of these oxidation pathways may offer a novel approach to preserve mitochondrial functions, protect telomere integrity, and maintain T cell survival, which can be exploited to combat various immune aging‐associated diseases.

## Graphical abstract

Oxidative stress caused by excess reactive oxygen species (ROS) accelerates telomere erosion and mitochondrial injury, leading to impaired cellular functions and cell death. By selectively producing singlet ^1^O_2_ to a single organelle, Wang and Lu et al. demonstrate that oxidative stress induces dual injury in T cells via crosstalk between telomeres and mitochondria, and also explore potential molecules and pathways that promote this dual‐damage.

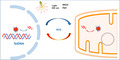

## 1 INTRODUCTION

T cells play a key role in controlling infectious and inflammatory disease, however, how T cell functions are dysregulated under these aberrant conditions remains poorly understood. Recent studies have highlighted the importance of shortened telomeres and impaired mitochondria in the age‐related decline of cellular functions in the elderly (Buck et al., 2016; Kirkwood, 2005; Ron‐Harel et al., 2015; Tedone et al., 2019). Notably, this natural aging phenotype is recapitulated in T cells derived from individuals with chronic viral infection (e.g., hepatitis C virus [HCV] or human immunodeficiency virus [HIV]) or inflammation (e.g., rheumatoid arthritis [RA]), which also exhibit shortened telomeres and impaired mitochondria (Blanco et al., 2015; Costenbader et al., 2011; Galluzzi et al., 2017; Lee & Bae, 2018; Lopez‐Armada et al., 2013; Younes et al., 2018; Zhao et al., 2018, 2019, 2021). To date, how telomeres and mitochondria are dysregulated to drive T cell aging and dysfunction during infection and/or inflammation remains largely unknown and warrants further investigation.

Telomere integrity protects the ends of chromosomes and preserves genomic stability, whereas telomere erosion is a hallmark of cell aging that promotes cell dysfunction or apoptosis (Arkus, 2005; Arnoult & Karlseder, 2015; Carneiro et al., 2016; Cavanagh et al., 2012). We have recently shown accelerated telomere erosion and mitochondrial dysfunction in T cells from virus‐infected individuals, indicating excessive cell proliferative turnover or increased irreparable DNA damage (Cao et al., 2020; Dang et al., 2020; Ji et al., 2019; Khanal et al., 2020; Nguyen et al., 2018; Schank et al., 2020, 2021; Zhao et al., 2018, 2019, 2021). Additionally, we have discovered that telomeric DNA damage induces a profound p53‐associated inhibition of the master mitochondrial regulators, peroxisome proliferator‐activated receptor gamma coactivator 1alpha (PGC‐1α) or mitochondrial transcription factor A (mtTFA), which is associated with mitochondrial compromise due to impaired oxidative phosphorylation and ATP generation (Schank et al., 2020, 2021; Zhao et al., 2021). Nevertheless, how telomeres are damaged and what the impacts of telomeric DNA damage are on mitochondrial functions during the T cell aging process in the setting of chronic infection and/or inflammation remain unclear.

Mitochondria are energy powerhouse organelles that critically affect cellular activities and viability (Buck et al., 2016; Ron‐Harel et al., 2015). Recently, we have found that T cells from virus‐suppressed HIV subjects exhibit not only shortened telomeres but also compromised mitochondrial functions, as evidenced by impaired mitochondrial respiration and ATP production upon T cell receptor (TCR) stimulation (Zhao et al., 2021). Importantly, we found that mitochondrial dysfunction increases the generation of ROS, inducing telomeric DNA damage, since telomeres are enriched with triple guanine repeats (TTAGGG), which are extremely sensitive to ROS‐mediated genotoxicity during oxidative stress (Schank et al., 2020). Questions that remain unanswered include: (1) how are mitochondrial functions dysregulated and does telomeric DNA damage trigger mitochondrial dysfunction in T cells; and (2) what are the effects of mitochondrial dysfunction on telomere integrity and T cell aging in inflammatory and infectious diseases.

Reactive oxygen species (ROS) are generated endogenously as natural byproducts of cell metabolism through oxidative phosphorylation in mitochondria (Devasagayam et al., 2004; Lonkar & Dedon, 2011) or exogenously via induction by diverse environmental exposures (Poljsak & Fink, 2014). When ROS generation exceeds cellular ROS scavenging capacity or antioxidant safeguards, oxidative stress occurs and contributes to the pathogenesis of numerous diseases, including cancer, inflammation, and age‐related cardiovascular, pulmonary, and neurodegenerative diseases (Hegde et al., 2012; Malinin et al., 2011; Reuter et al., 2010). Indeed, previous studies have shown that oxidative stress leads to inhibition of cell proliferation and accumulation of telomere DNA damage and telomere shortening; whereas reducing oxidative stress by antioxidants decreases the telomere shortening rate and increases the replicative life span (von Zglinicki, 2002; von Zglinicki et al., 1995, 2000).

It has been suggested that oxidative stress caused by excess ROS accelerates telomere erosion and mitochondrial injury (Ahmed & Lingner, 2018; Barnes et al., 2019; Blanco et al., 2015; Costenbader et al., 2011; Galluzzi et al., 2017; Lee & Bae, 2018; Lopez‐Armada et al., 2013; Nguyen et al., 2018; Younes et al., 2018; Zhao et al., 2018, 2019, 2021). A dynamic feedback loop between telomeric and mitochondrial injury mediated by ROS has been described previously (Passos et al., 2010). To phenotypically confirm the existence of a two‐way interaction between mitochondria and telomeres in T cells following oxidative injury, we employed a highly innovative tool that selectively produces a common oxidative reaction at telomeres or mitochondria and then investigated its dual‐effect on telomere integrity and mitochondrial functions in T cells. This novel technology involves tagging a specific cellular compartment protein with fluorogen‐activating peptide (FAP) and fluorescent mCerulean (mCer) to visualize the expression of the fusion protein in a specific cellular compartment (Fouquerel et al., 2016; Telmer et al., 2015; Wang et al., 2015). FAPs have a high affinity for the photosensitizer dye di‐iodinated malachite green (MG2I), which produces a single oxygen (^1^O_2_) only upon FAP binding and subsequent excitation with a high‐intensity 660‐nm light‐emitting diode, thus triggering targeted oxidative damage without causing off‐target damage (Fouquerel et al., 2019; He et al., 2016). Guanine is the most commonly oxidized nucleotide base (Steenken, 1997), and telomeric TTAGGG repeats are preferred sites for conversion of G to 8‐oxoguanine (8‐oxoG) lesions in the human genome (Henle et al., 1999; Oikawa et al., 2001). Therefore, ^1^O_2_ primarily generates 8‐oxoG upon reaction with DNA (Ravanat et al., 2001) and has a very short half‐life (100 nanoseconds) in live cells (Agnez‐Lima et al., 2012), allowing highly localized oxidative reactions.

To elucidate the mechanisms by which oxidative stress triggers telomeric and mitochondrial dysfunction and to characterize how their crosstalk leads to T cell aging during inflammation, we employed a chemoptogenetic tool that selectively produces oxidative damage only at telomeres or mitochondria through local production of ^1^O_2_ without causing oxidative damage elsewhere inside the cell. We demonstrate that selective ^1^O_2_ targeting of telomeres drives not only telomere erosion but also mitochondrial compromise. In contrast, we find that specific ^1^O_2_ targeting of mitochondria causes not only mitochondrial dysfunction but also telomere attrition. These dual‐damage effects are dose‐ and time‐dependent, that is, repeating this oxidative stress over time induces greater oxidative damage and cellular crisis, leading to more T cell apoptotic death. We also explored potential molecules and pathways that promote this dual damage via crosstalk between mitochondria and telomeres in T cells during oxidative stress.

## 2 RESULTS

### 2.1 Selective oxidation at telomeres induces telomeric DNA damage and T cell apoptosis

**FIGURE 1:**
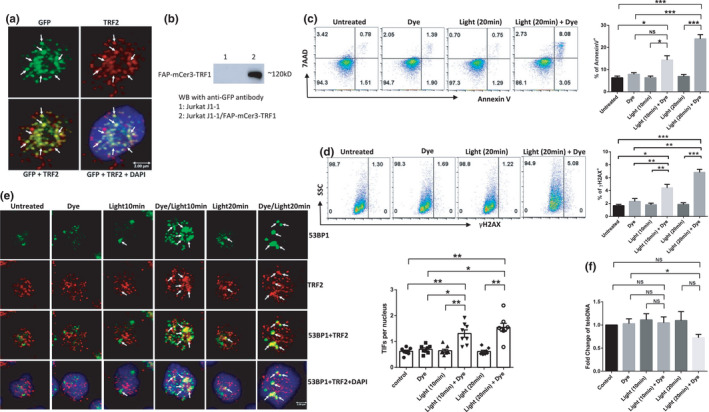
Oxidative stress at telomere induces telomeric DNA damage and apoptosis in T cells. (a) Confocal microscopy images showing FAP‐mCer3‐TRF1 (Green) colocalization with shelterin protein TRF2 (Red) in the nucleus (DAPI Blue) in J1.1/FAP‐mCer3‐TRF1 cells. (b) Western blot of FAP‐mCer3‐TRF1 fusion protein (~120 kD) in Jurkat J1.1 cells and J1.1/FAP‐mCer3‐TRF1 cells. (c) Flow cytometry analysis of J1.1/FAP‐mCer3‐TRF1 cell apoptosis after MG2I treatment and light exposure for 10 min/day or 20 min/day for 3 days. Representative pseudocolor plots and summary data of Annexin V^+^ cells from 6 independent experiments are shown (*p < 0.05; ***p < 0.001, one‐way ANOVA). (d) Flow cytometry analysis of DNA damage marker γH2AX in J1.1/FAP‐mCer3‐TRF1 cells after MG2I treatment and light exposure for 10 min/day or 20 min/day for 3 days. Representative pseudocolor plots and summary data of γH2AX^+^ cells from 6 independent experiments are shown (*p < 0.05; **p < 0.01; ***p < 0.001, one‐way ANOVA). (e) Telomeric DNA damage in J1.1/FAP‐mCer3‐TRF1cells after MG2I treatment and light exposure for 10 min/day or 20 min/day for 3 days versus control cells was analyzed by confocal microscopy for colocalization of 53BP1 (Green) and TRF2 (Red), which is located in the nucleus (DAPI Blue). Representative images and summary data for the number of TIFs per nucleus counted with 50 cells per experiment from 8 independent experiments (*p < 0.05; **p < 0.01, one‐way ANOVA). (f) teloDNA versus nuDNA content measured by real‐time qPCR in Jurkat J1.1 cells after MG2I treatment and light exposure for 10 min/day or 20 min/day for 3 days versus control cells. Data from 5 independent experiments are shown (*p < 0.05; NS = no significance, one‐way ANOVA)

Telomeric repeat‐binding factor 1 (TRF1) is a shelterin protein that directly binds to telomeric DNA to form a nucleoprotein complex at chromosome ends. To selectively induce oxidative stress at telomeres in T cells, TRF1 was tagged with FAP and mCer in a construct (FAP‐mCer3‐TRF1) that was stably transfected into Jurkat J1.1 cells. After immunostaining with rabbit anti‐GFP (recognizing mCer3) and mouse anti‐telomeric repeat‐binding factor 2 (TRF2) antibodies, we performed confocal microscopic analysis of FAP‐mCer3‐TRF1 expression in the transfected cells. Figure 1a shows that FAP‐mCer3‐TRF1 (Green) colocalized with shelterin protein TRF2 (Red), as demonstrated by the overlapped signals (yellow), which are all located in the nucleus (Blue DAPI), indicating that the fusion protein is specifically expressed at telomeres. Western blotting revealed that the FAP‐mCer3‐TRF1 fusion protein was only expressed in J1.1/FAP‐mCer3‐TRF1 cells but not in parental J1.1 cells (Figure 1b).

A previous study has shown 8‐oxoG formation at telomeres in HeLa cells using the approach described above (Fouquerel et al., 2019). To determine the biological effect of targeted telomeric 8‐oxoG base damage in T cells, J1.1/FAP‐mCer3‐TRF1 cells were exposed to 200 nM of MG2I and/or 660 nm LED light for 10 or 20 min each day. The cells were harvested at 24 h after three treatments for the measurement of cellular apoptosis by Annexin V/7AAD staining. As shown in Figure 1c (representative pseudocolor plots and summary data of the percentage (%) of Annexin V^+^ cells from flow cytometry), both 10 min/day and 20 min/day of light plus dye exposure for 3 days induced cell apoptosis, but 20 min/day of light plus dye treatments led to a very significant increase of cell apoptosis compared to the untreated, only dye, or only light‐exposed cells. Similar trends were also observed in early apoptotic (Annexin V^+^/7AAD^−^) and late apoptotic cells (Annexin V^+^/7AAD^+^) after 10 min/day or 20 min/day light plus dye exposure for 3 days (Figure S1a).

Following genomic insults, histone variant H2AX is phosphorylated on serine (S139) at the DNA damage site, forming γH2AX that subsequently recruits other DNA damage and repair proteins to trigger DNA damage response (DDR) (Kuo & Yang, 2008). To determine whether DDR plays a role in cellular apoptosis during telomeric oxidative stress, we measured γH2AX expression in J1.1/FAP‐mCer3‐TRF1 cells and found remarkably increased levels of γH2AX after both 10 min/day or 20 min/day of light/MG2I treatments for 3 days (Figure 1d). The γH2AX level was increased with one and two treatments, but two treatments led to a significant increase in γH2AX levels after both 10 min and 20 min exposures (Figure S1b), indicating a dose‐ and time‐dependent effect on telomeric DNA damage in T cells with oxidative stress.

In conjunction with H2AX phosphorylation, 53BP1 is recruited to the DNA damage site (including in the vicinity of telomeric breaks), acting as a docking site for other adaptor proteins to form microscopically visible nuclear foci (DNA damage foci). Thus, identifying dysfunctional telomere‐induced foci (TIF) is typically regarded as a hallmark of telomeric DDR (Rothkamm et al., 2015; Takai et al., 2003). Confocal microscopy analysis revealed that DNA damage was localized at telomeres, as shown by the colocalization of DNA damage marker 53BP1 with telomere shelterin protein TRF2. The number of TIFs per nucleus in J1.1/FAP‐mCer3‐TRF1 cells was significantly increased after both 10 min/day and 20 min/day with 3 days of light exposure plus MG2I treatments when compared to the controls (Figure 1e). Additionally, we also compared telomeric DNA (telDNA) to nuDNA (36B4 DNA, a single‐copy gene that codes for a ribosomal protein) content using real‐time qPCR and found reduced, but not statistically different, telomere copy numbers in T cells exposed to MG2I and light for 20 min/day for 3 days, when compared to the controls (Figure 1f).

Taken together, these results suggest that selective oxidative stress at telomere leads to a dose‐ and time‐dependent increase in telomeric DNA damage and T cell apoptosis.

### 2.2 Oxidation stress at mitochondria induces mitochondrial dysfunction and T cell apoptosis

**FIGURE 2:**
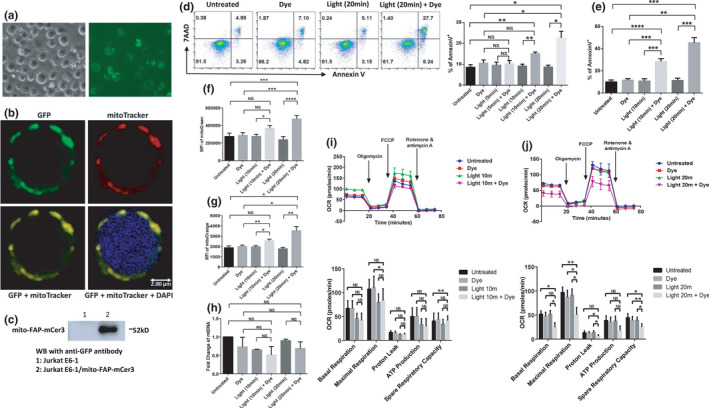
Oxidative stress at mitochondria induces mitochondrial dysfunction and cell apoptosis. (a) Bright field and fluorescent microscopy imaging of mito‐FAP‐mCer3 stably transfected Jurkat E6‐1 cells. (b) Confocal microscopy images of the mito‐FAP‐mCer3 fusion protein (Green) and MitoTracker Red CMXRos (Red) after immunostaining of stably transfected and sorted Jurkat E6‐1 cells. The nucleus (Blue) was counter‐stained with DAPI. (c) Western blot of the mito‐FAP‐mCer3 fusion protein (~52 kD) in Jurkat E6‐1 cells and E6‐1/mito‐FAP‐mCer3 cells. (d) Flow cytometry analysis of E6‐1/mito‐FAP‐mCer3 cell apoptosis after MG2I treatment and light exposure for 5, 10, or 20 min/day for one day. Representative pseudocolor plots and summary data of Annexin V^+^ cells from 8 independent experiments are shown (*p < 0.05; **p < 0.01; NS = no significance, one‐way ANOVA). (e) Summary data of cell apoptosis from 7 independent experiments after MG2I treatment and light exposure 10 min/day or 20 min/day for 3 days (**p < 0.01; ***p < 0.001; ****p < 0.0001, one‐way ANOVA). (f) MFI of MG in E6‐1/mito‐FAP‐mCer3 cells after MG2I treatment and/or light exposure 3 times. Summary data from 8 independent experiments are shown (*p < 0.05; ***p < 0.001; ****p < 0.0001; NS = no significance, one‐way ANOVA). (g) MFI of MO in E6‐1/mito‐FAP‐mCer3 cells after MG2I treatment and/or light exposure 3 times. Summary data from 8 independent experiments are shown (*p < 0.05; **p < 0.01; NS = no significance, one‐way ANOVA). (h) mtDNA level relative to nuDNA content experiments after MG2I treatment and light exposure 10 min/day or 20 min/day for 3 days. Summary data from 3 independent experiments are shown (NS = no significance, one‐way ANOVA). (i,j) OCR for basal respiration, maximal respiration, proton leak, ATP production, and spare respiratory capacity in E6‐1/mito‐FAP‐mCer3 cells after MG2I treatment and 10 (i) or 20 min (j) light exposure for one time. Summary data from 4 (10 min) and 5 (20 min) independent experiments are shown (*p < 0.05; **p < 0.01; NS = no significance, one‐way ANOVA)

As the primary source of cellular ROS, mitochondria are substantially involved in oxidative stress (Marchi et al., 2012). To investigate the specific effects of oxidative stress at mitochondria, we employed a chemoptogenetic tool that involves tagging the mitochondrial COX IV‐COX VIII sequences (Mito) to FAP‐mCer3 sequences in an expression plasmid (pCDNA3.1/COX IV‐COX VIII‐dL5‐2XG4S‐mCer3) (Wang et al., 2015), which was transfected into Jurkat E6‐1 cells. The E6‐1/mito‐FAP‐mCer3 stable cell line was established by G418 selection and mCer3^+^ cell sorting. Using an EVOS FL cell imaging system, the green fluorescent signals from mito‐FAP‐mCer3 fusion protein were detected within the cells, located in the cytoplasm as outer‐rings around the cell membrane (Figure 2a). Using MitoTracker Red CMXROS staining and immunostaining with rabbit anti‐GFP, and imaging with the Leica DMI8 confocal system, we further confirmed that the expression of mito‐FAP‐mCer3 fusion protein (green) was localized in the mitochondria (red). The nuclei were labeled with DAPI (blue) (Figure 2b). Western blot analysis revealed that mito‐FAP‐mCer3 fusion protein was only expressed in the transfected cells but not in the parental Jurkat E6‐1 cells (Figure 2c).

To determine if cell apoptosis is induced by mitochondrial oxidative stress, E6‐1/mito‐FAP‐mCer3 cells were exposed to 200 nM of MG2I and 660 nm LED light for 5, 10, or 20 min. After removing MG2I and incubating in RPMI 1640 medium for 24 h, the cells were harvested for apoptosis detection. As shown in Figure 2d, a significant increase in overall apoptosis (Annexin V^+^) was observed with MG2I treatment plus 10 min or 20 min of light exposure when compared with untreated cells, only MG2I treatment, or only light exposure. Treatment with MG2I plus 5 min/day light exposure for 1 day did not induce significant cell apoptosis in E6‐1/mito‐FAP‐mCer3 cells (Figure 2d and Figure S1c). Late apoptosis (Annexin V^+^/7AAD^+^) but not early apoptosis (Annexin V^+^/7AAD^−^) was observed in cells with MG2I treatment plus 20 min/day light exposure for 1 day compared with the control groups (Figure S1c). To determine if this effect is time‐dependent, we treated the cells with MG2I plus 10 min or 20 min of light exposure each day for three consecutive days and observed a dramatic increase in total, early, and late apoptosis (Figure 2e and Figure S1d). These data indicate that mitochondrial oxidative stress induces dose‐ and time‐dependent cell apoptosis.

To investigate the effects of oxidative stress on mitochondrial functions, we measured MitoTracker Green (MG), MitoTracker Orange (MO), mitochondrial DNA (mtDNA) content, oxygen consumption rate (OCR), and extracellular acidification rate (ECAR). MG is a green fluorescent mitochondrial stain that selectively binds to the free thiol groups of cysteine residues enriched in mitochondrial proteins regardless of mitochondrial membrane potential and is commonly used to represent mitochondrial mass (Agnello et al., 2008; Cottet‐Rousselle et al., 2011; Xiao et al., 2016; Zhou et al., 2011). Here, we treated E6‐1/mito‐FAP‐mCer3 cells with MG2I and exposed to both 10 min/day and 20 min/day of light for 3 days, followed by measuring the mean fluorescence intensity (MFI) of MG by flow cytometry. As shown in Figure 2f, MG2I treatment plus 10 min/day light exposure slightly increased MFI of MG, but MG2I treatment plus 20 min/day light exposure significantly increased the MFI of MG compared with the untreated controls, dye only, or light only treatment.

MitoTracker Orange is an orange fluorescent dye that stains mitochondria in live cells and its accumulation is dependent upon membrane potential. MO is oxidized by molecular oxygen in actively respiring cells, and therefore it allows for measuring the mitochondrial membrane potential and oxidative phosphorylation (Agnello et al., 2008). To investigate mitochondrial oxidation, we measured MO in E6‐1/mito‐FAP‐mCer3 cells treated with MG2I and exposed to light for 10 min/day or 20 min/day for 3 days using flow cytometry. These treatments dramatically increased the MFI of MO (Figure 2g), indicating compromised mitochondrial membrane potential and oxidative phosphorylation during oxidative stress.

In addition to MG and MO, we measured mtDNA relative to nuDNA content using real‐time qPCR. As shown in Figure 2h, the cells treated with MG2I and exposed to light did not elicit significant changes in mtDNA copy numbers, indicating that mtDNA integrity is relatively stable and not affected by selective mitochondrial oxidative stress.

To assess mitochondrial respiration, we conducted cell Mito stress tests using a Seahorse XFp analyzer, an ideal approach to investigate mitochondrial OCR and ECAR, including mitochondrial basal and maximal respiration, proton leak, ATP production, and spare respiratory capacity upon cellular challenges (Divakaruni et al., 2014; Leung & Chu, 2018). As shown in Figure 2i,j, cells treated with MG2I plus light exposure for 10 or 20 min one time led to significant decreases in maximal mitochondrial respiration and spare respiratory capacities compared with the untreated controls, and this effect was more evident after 20 min light exposure. Basal glycolysis, as indicated by the ECAR, was significantly decreased in the cells treated with MG2I plus 20 min light exposure compared with only dye and only light‐exposed cells (Figure S1f), but not in the cells treated with MG2I plus 10 min light exposure (Figure S1e), compared with the controls.

In essence, these results suggest that upon mitochondrial oxidative stress, T cells exhibit compromised mitochondrial functions, leading to cell apoptotic death.

### 2.3 Selective oxidative stress induces dual damage to telomeres and mitochondria in T cells

**FIGURE 3:**
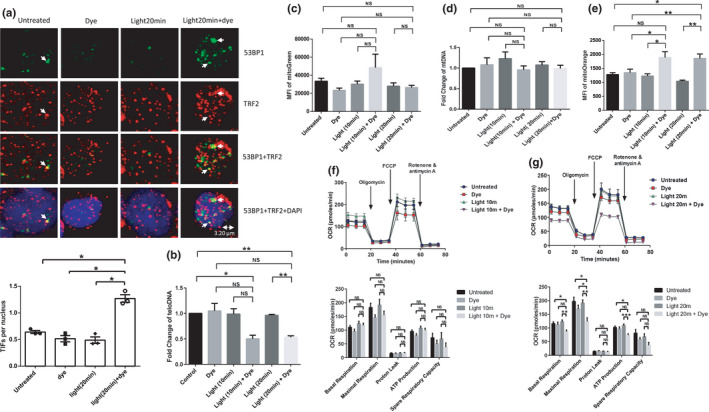
Dual damage at telomeres and mitochondria in T cells. (a) Telomeric DNA damage in E6.1/mito‐FAP‐mCer3 cells after treatment with MG2I and light exposure for 20 min/day for 3 days was analyzed by confocal microscopy for colocalization of 53BP1 (Green) and TRF2 (Red), which is located in the nucleus (DAPI Blue). Representative images and summary data of TIFs per nucleus counted with 50 cells per experiment from 3 independent experiments (*p < 0.05, one‐way ANOVA). (b) Summary data of teloDNA level versus nuDNA content was measured by real‐time qPCR in E6‐1/mito‐FAP‐mCer3 cells after MG2I treatment and/or light exposure for 10 min/day or 20 min/day for 5 days. Data are from 4 independent experiments (*p < 0.05; **p < 0.01; NS = no significance, one‐way ANOVA). (c) MFI of MG change in J1.1/FAP‐mCer3‐TRF1 cells after MG2I treatment and/or light exposure for 10 min/day or 20 min/day for 3 days. Data are from 6 independent experiments (NS = no significance, one‐way ANOVA). (d) mtDNA level relative to nuDNA content in J1.1/mito‐FAP‐mCer3 cells after MG2I treatment and/or light exposure for 10 min/day or 20 min/day for 5 days. Summary data from 7 independent experiments are shown (NS = no significance, one‐way ANOVA). (e) Summary data for MFI of MO in J1.1/FAP‐mCer3‐TRF1 cells after MG2I treatment and/or light exposure for 10 min/day or 20 min/day for 3 days were significantly increased after MG2I treatment and light exposure from 6 independent experiments (*p < 0.05; **p < 0.01; NS = no significance, one‐way ANOVA). (f,g) OCR of J1.1/FAP‐mCer3‐TRF1 cells after MG2I treatment and light exposure for 10 min/day or 20 min/day for 3 days. Oligomycin, FCCP, rotenone, and antimycin A were sequentially injected into each well. Summary data from 6 (10 min) and 8 (20 min) independent experiments are shown (*p < 0.05; **p < 0.01; ***p < 0.001; NS = no significance, one‐way ANOVA)

To determine whether selective oxidative stress at mitochondria can cause telomere attrition, we treated E6‐1/mito‐FAP‐mCer3 cells with or without MG2I and/or light exposure for 20 min/day for 3 days, followed by confocal microscopy and real‐time qPCR analysis. As shown in Figure 3a, a significant increase in TIFs was observed in MG2I plus light‐treated cells compared with the control groups. Correspondingly, telDNA content in MG2I plus light‐treated cells was significantly reduced after 20 min/day treatment for 5 days compared with the untreated and only light controls (Figure 3b).

We also measured mitochondrial functions in J1.1/FAP‐mCer3‐TRF1 cells exposed to MG2I and/or light. While the MFI of MG and mtDNA content were not affected (Figure 3c,d), the MFI of MO—reflecting the mitochondrial membrane potential and oxidative phosphorylation—was significantly increased compared with the controls (Figure 3e). In addition, the mitochondrial maximal respiration was significantly reduced in cells exposed to MG2I plus light for 20 min/day, but not 10 min/day, for 3 days when compared with the controls (Figure 3f,g). There was little difference in the basal glycolysis among the cells treated with MG2I plus 10 min/day or 20 min/day light plus dye exposure compared with the controls (Figure S1g,h).

Taken together, these results suggest that selective oxidative stress at mitochondria or telomeres induces dual damage at both the mitochondria and nuclei, implying crosstalk between these two organelles in T cells undergoing oxidative stress (Zheng et al., 2019).

### 2.4 Inhibition of ROS production reduces cellular injury and apoptosis during oxidative stress

**FIGURE 4:**
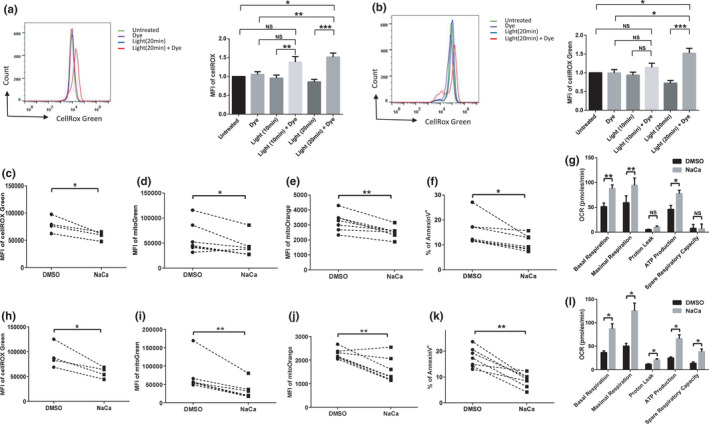
Inhibition of ROS reduces apoptosis and mitochondrial function in T cells undergoing oxidative stress. (a) Overlaid histogram and summary data for MFI of cellROX green in J1‐1/FAP‐mCer3‐TRF1 cells after MG2I treatment and/or light exposure for 10 min/day or 20 min/day for 3 days. Data are from 9 independent experiments (*p < 0.05; **p < 0.01; ***p < 0.001, NS = no significance, one‐way ANOVA). (b) Overlaid histogram and summary data for MFI of cellROX green in E6‐1/mito‐FAP‐mCer3 cells after MG2I treatment and/or light exposure 10 min/day or 20 min/day for 3 days. Data are from 8 independent experiments (*p < 0.05; ***p < 0.001, NS = no significance, one‐way ANOVA). (c) Summary data for MFI of cellROX green in J1.1/FAP‐mCer3‐TRF1 cells after MG2I treatment and light exposure for 20 min/day for 3 days in the presence of NaCa or DMSO. Data are from 4 independent experiments (*p < 0.05, paired *t*‐test). (d) Summary data for MFI of mitoGreen in J1.1/FAP‐mCer3‐TRF1 cells after MG2I treatment and light exposure for 20 min/day for 3 days in the presence of NaCa or DMSO. Data are from 7 independent experiments (*p < 0.05, paired *t*‐test). (e) Summary data for MFI of mitoOrange in J1.1/FAP‐mCer3‐TRF1 cells after MG2I treatment and light exposure for 20 min for 3 times in the presence of NaCa or DMSO. Data are from 7 independent experiments (**p < 0.01, paired *t*‐test). (f) Apoptosis of J1.1/FAP‐mCer3‐TRF1 cells after MG2I treatment and light exposure for 20 min/day for 3 days in the presence of NaCa or DMSO. Data are from 7 independent experiments (*p < 0.05, paired *t*‐test). (g) OCR of J1.1/FAP‐mCer3‐TRF1 cells MG2I treatment and light exposure for 20 min/day for 3 days in the presence of NaCa or DMSO. Oligomycin, FCCP, and rotenone, and antimycin A were sequentially injected into each well. Summary data for basal respiration, maximal respiration, proton leak, ATP production, and spare respiratory capacity from 3 independent experiments are shown (*p < 0.05; **p < 0.01; NS = no significance, paired *t*‐test). (h) Summary data for MFI of cellROX green in E6‐1/mito‐FAP‐mCer3 cells after MG2I treatment and light exposure for 20 min for 20 min/day for 3 days in the presence of NaCa or DMSO. Data are from 4 independent experiments (*p < 0.05, paired *t*‐test). (i) Summary data for MFI of mitoGreen in E6‐1/mito‐FAP‐mCer3 cells after MG2I and light exposure for 20 min/day for 3 days in the presence of Naca or DMSO. Data are from 7 independent experiments (**p < 0.01, paired *t*‐test). (j) Summary data for MFI of mitoOrange in E6‐1/mito‐FAP‐mCer3 cells after MG2I treatment and light exposure for 20 min/day for 3 days in the presence of Naca or DMSO. Data are from 7 independent experiments (**p < 0.01, paired *t*‐test). (k) Apoptosis in E6‐1/mito‐FAP‐mCer3 cells after MG2I treatment and light exposure for 20 min/day for 3 days in the presence of NaCa or DMSO. Data are from 7 independent experiments (**p < 0.01, paired *t*‐test). (l) OCR in E6‐1/mito‐FAP‐mCer3 cells after MG2I treatment and light exposure for 20 min/day for 3 days in the presence of NaCa or DMSO. Oligomycin, FCCP, rotenone, and antimycin A were sequentially injected into each well. Summary data for basal respiration, maximal respiration, proton leak, ATP production, and spare respiratory capacity from 3 independent experiments are shown (*p < 0.05, paired *t*‐test)

To determine the mechanism underlying these biologic effects on mitochondria and telomeres, we measured intracellular ROS production in treated cells using a fluorescence‐based ROS detection method (cellROX Green). As shown in Figure 4a,b, the MFI of cellROX green was slightly increased in both J1.1/FAP‐mCer3‐TRF1 (Figure 4a) and E6‐1/mito‐FAP‐mCer3 cells (Figure 4b) exposed to MG2I plus light for 10 min each day for 3 days, but MG2I plus 20 min light/day led to a dramatical increase of the MFI of cellROX green in both of the two cells compared with the untreated controls, dye only, or light only treatment. The data suggest an important role for ROS in inducing these cellular injuries.

N‐acetylcysteine amide (NaCa) is a membrane penetrating antioxidant with anti‐inflammatory activity and is commonly used to reduce cellular ROS production (Kim et al., 2013; Wang et al., 2018). To determine the role of ROS in telomere injury‐mediated mitochondrial damage, J1.1/FAP‐mCer3‐TRF1 cells were exposed to MG2I and/or light for 20 min/day for 3 days in the presence of NaCa or DMSO, followed by measuring mitochondrial functions and cell apoptosis. As shown in Figure 4c–e, the MFIs of ROS, MG, and MO were significantly reduced in NaCa‐treated cells compared to the DMSO‐treated control. Moreover, total, early, and late cell apoptosis were also reduced in the NaCa‐treated group (Figure 4f, Figure S2a). In addition, mitochondrial basal and maximal respiration, as well as ATP production, were improved by the NaCa treatment (Figure 4g). Correspondingly, we performed the same experiments using E6‐1/mito‐FAP‐mCer3 cells exposed to MG2I and light in the presence of NaCa or DMSO and observed the same results—that is, blocking ROS production by NaCa significantly reduced ROS production (Figure 4h), the MFI of MG (Figure 4i), the MFI of MO (Figure 4j), and cell apoptosis (Figure 4k, Figure S2b) and improved mitochondrial OCR (Figure 4l) when compared to the DMSO‐treated control.

Taken together, these results suggest that selective oxidative stress at telomeres or mitochondria can induce ROS production in T cells and that blocking ROS generation during oxidative stress can improve mitochondria or telomere functions and reduce cell apoptosis.

### 2.5 Identification of molecular pathways involved in telomeric DNA damage and mitochondrial injury during oxidative stress

**FIGURE 5:**
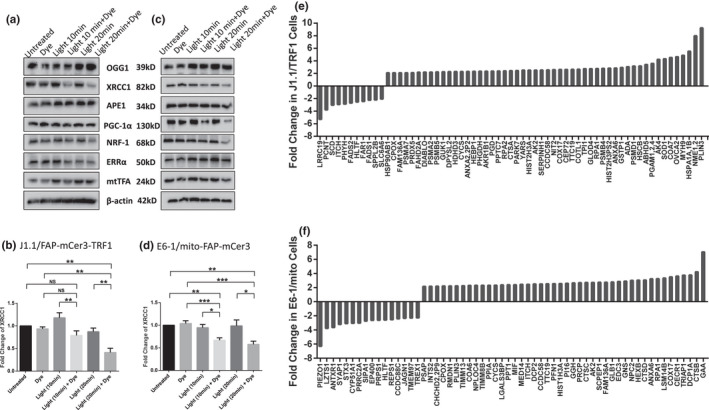
Western blot and mass spectrometry analysis. (a, c) Representative western blots of the DNA repair proteins (OGG1, XRCC1, and APE1), the PGC‐1α network proteins (PGC‐1α, NRF‐1, ERRα), and mtTFA in J1.1/FAP‐mCer3‐TRF1 cells (a) and E6‐1/mito‐FAP‐mCer3 cells (c) with or without MG2I and light exposure for 10~20 min/day for 3 days. (b, d) Summary data of XRCC1 protein levels in J1.1/FAP‐mCer3‐TRF1 cells from 5 independent experiments (b) and E6‐1/mito‐FAP‐mCer3 cells from 8 independent experiments (d) with or without MG2I and light exposure for 10~20 min/day for 3 days (*p < 0.05; **p < 0.01; ***p < 0.001; NS = no significance, one‐way ANOVA). (e) Proteomic analysis of protein changes due to oxidative stress at telomeres in J1.1/FAP‐mCer3‐TRF1 cells treated with MG2I and light exposure for 20 min/day for 3 days compared to the controls. (f). Changes in protein levels due to oxidative stress at mitochondria in E6‐1/mito‐FAP‐mCer3 cells treated with MG2I and light exposure for 20 min/day for 3 days compared to the controls, analyzed by LC‐MS

While we did not observe any change in mtDNA levels, we found alterations in telomeric DNA content, in both cell lines under targeted oxidative stress, suggesting that circular mtDNA are more genetically stable than linear telomeric DNA. The main machinery for DNA repair of oxidized bases is via the base excision repair (BER) pathway. Notably, increased oxidative stress strongly correlates with a decrease in BER in the aging process and age‐related neurodegenerative diseases (Leandro et al., 2015). Many DNA damage repair‐related enzymes have been implicated in the BER pathway, including 8‐Oxoguanine glycosylase 1 (OGG1) (de Souza‐Pinto et al., 2001), apurinic/apyrimidinic (AP) endonuclease (APE1) (Wallace, 2013), and X‐ray repair cross‐complementing protein 1 (XRCC1) (Ellenberger & Tomkinson, 2008). To determine whether the BER pathway is involved in the oxidative stress‐induced teloDNA reduction, we treated both E6‐1/mito‐FAP‐mCer3 cells and J1.1/FAP‐mCer3‐TRF1 cells with or without dye plus light exposure for 10~20 min/day for 3 days, followed by Western blot analysis to measure the expression of these signaling molecules 24 h after the last treatment. Intriguingly, selective oxidative stress at telomeres (Figure 5a,b) and mitochondria (Figure 5c,d) significantly suppressed XRCC1, but not OGG1 and APE1 levels, in the treated cells compared to the controls—similar to our previous observations in primary CD4^+^ T cells treated with a telomere‐targeting drug KML001 (Schank et al., 2020).

We have recently shown that PGC‐1α (a master mitochondrial regulator) is suppressed in human CD4^+^ T cells derived from virally (HIV or HCV) infected patients and CD4^+^ T cells from healthy subjects following treatment with KML001 (Schank et al., 2020; Zhao et al., 2021). To determine whether the PGC‐1α pathway is impacted by the selective oxidative stress, we performed Western Blotting to determine the expression of PGC‐1α and its downstream transcription factors, including nuclear respiratory factor 1 (NRF‐1) and estrogen‐related receptor alpha (ERRα), in J1.1/FAP‐mCer3‐TRF1 cells (Figure 5a) and E6‐1/mito‐FAP‐mCer3 cells (Figure 5c) treated with MG2I and 10~20 min/day light exposure for 3 days. As shown in Figure S2c,d, PGC‐1α protein levels were slightly decreased in E6‐1/mito‐FAP‐mCer3 cells treated with MG2I and 20 min/day light exposure for 3 days, but there were no differences in the protein levels of PGC‐1α in J1.1/FAP‐mCer3‐TRF1 cells, compared to the controls. We also examined the expression of mtTFA—another mitochondrial master regulator that is significantly inhibited in CD4^+^ T cells derived from individuals with chronic HIV (Zhao et al., 2021) or HCV (unpublished observations) infection—and no significant differences were observed in mtTFA levels in either group of cells after the treatment with MG2I and 10~20 min/day light exposure for 3 days (Figure 5a,c).

To further elucidate the molecular mechanisms underlying mitochondrial dysregulation in the setting of oxidative stress, mitochondrial proteins from both J1.1/FAP‐mCer3‐TRF1 and E6‐1/mito‐FAP‐mCer3 cells treated with or without MG2I and light exposure 20 min/day for 3 days were isolated and analyzed by liquid chromatography‐tandem mass spectrometry (LC‐MS). A total of 1,023 known mitochondrial proteins were screened using this approach. We identified 60 proteins (44 up‐regulated and 16 down‐regulated) in E6‐1/mito‐FAP‐mCer3 cells, and 63 proteins (52 up‐regulated and 11 down‐regulated) in J1.1/FAP‐mCer3‐TRF1 cells, that exhibited >2‐fold change in cells treated with MG2I plus 20 min/day light exposure for 3 days compared to their respective controls (Figure 5e,f). Notably, 11 mitochondrial proteins were up‐regulated in both J1.1/FAP‐mCer3‐TRF1 and E6‐1/mito‐FAP‐mCer3 cells by selective oxidative stress, including cytochrome c oxidase 17 (COX17), tetratricopeptide repeat protein 19 (TTC19), cytochrome c (CYCS), replication protein A1 (RPA1), and Adenylate kinases 2 (AK2), all of which are known to play pivotal roles in regulating mitochondrial functions. Because many of these mitochondrial proteins were dysregulated by oxidative stress, their roles in regulating mitochondrial functions will be investigated further.

## 3 DISCUSSION

Excess ROS produced under oxidative stress during viral infection or inflammation can cause premature T cell aging (Blanco et al., 2015; Costenbader et al., 2011; Galluzzi et al., 2017; Lee & Bae, 2018; Lopez‐Armada et al., 2013; Schank et al., 2020; Younes et al., 2018; Zhao et al., 2021). Telomeres are essential for genomic stability (Arkus, 2005; Arnoult & Karlseder, 2015; Carneiro et al., 2016; Cavanagh et al., 2012) and mitochondria are the cellular powerhouse (Buck et al., 2016; Ron‐Harel et al., 2015). Previous studies have shown that DNA damage response (DDR) triggers mitochondrial dysfunction, leading to enhanced ROS activation via a linear signal transduction through the GADD45‐MAPK14 (p38MAPK)‐GRB2‐TGFBR2‐TGFβ pathway, and the ROS in turn replenish short‐lived DNA damage foci and maintain an ongoing DDR (Passos et al., 2010). Notably, TRF2ΔBΔM is a truncated dominant‐negative form of telomere‐capping factor TRF2, and its overexpression strips off endogenous TRF2 from telomeres and leads to progressive telomeric uncapping, and consequently, telomere damage‐mediated cell senescence (d'Adda di Fagagna et al., 2003; van Steensel et al., 1998), along with mitochondrial dysfunction and ROS production (Passos et al., 2010). ROS inhibition enhances mitochondrial membrane potential, and dual inhibition of ROS and mTOR can further reduce senescence‐induced mitochondrial dysfunction and DNA double‐strand breaks (Dalle Pezze et al., 2014). As a major driver of cellular senescence, DDR interacts with mitochondria to develop a senescent phenotype, and the cascade of ATM, Akt, and mTORC1 phosphorylation plays a key role as an effector of the DDR by promoting PGC‐1β‐dependent mitochondrial biogenesis and cellular senescence (Correia‐Melo et al., 2016).

**FIGURE 6:**
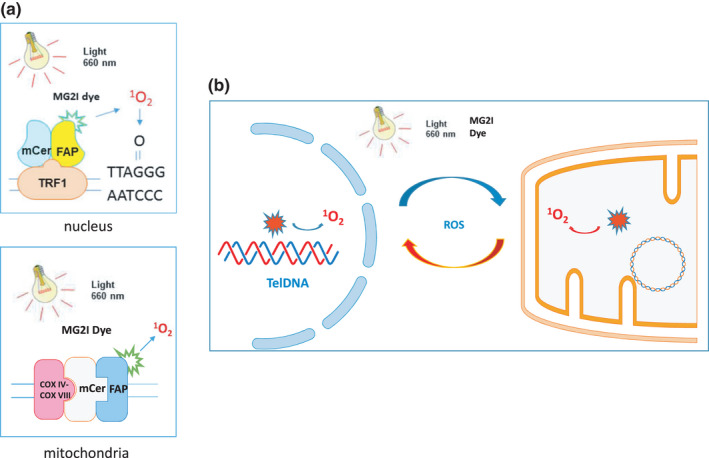
Model of the chemoptogenetic tool selectively inducing oxidative stress and causes dual damage at telomeres and mitochondria. (a) Schematic of the chemoptogenetic tools inducing single ^1^O_2_ production at telomeres and mitochondria upon exposure to MG2I dye and light. (b) Crosstalk between telomere and mitochondrial dual‐injury during oxidative stress

While telomere attrition and mitochondrial dysfunction are well‐known hallmarks of aging cells (Buck et al., 2016; Kirkwood, 2005; Ron‐Harel et al., 2015; Tedone et al., 2019), the mechanisms underlying the alterations of these aging markers, especially the role of oxidative stress in shortening telomere length and compromising mitochondrial functions in T cells, are incompletely understood and very challenging to study because ROS and induced 8‐oxoG have pleiotropic as well as epigenetic‐like effects on cellular functions (Devasagayam et al., 2004; Hegde et al., 2012; Lonkar & Dedon, 2011; Malinin et al., 2011; Poljsak & Fink, 2014; Reuter et al., 2010). In this study, we employed a novel chemoptogenetic tool (Agnez‐Lima et al., 2012; Fouquerel et al., 2016, 2019; He et al., 2016; Henle et al., 1999; Oikawa et al., 2001; Ravanat et al., 2001; Steenken, 1997; Telmer et al., 2015; Wang et al., 2015) that selectively produces a singlet ^1^O_2_ at telomeres or mitochondria and demonstrated that targeted oxidative stress accelerates telomere erosion as well as mitochondrial dysfunction. Interestingly, selective oxidation at telomeres not only induced telomeric DNA damage but also caused mitochondrial compromise, leading to cell crisis. Also, targeted oxidation at mitochondria caused telomere injury, resulting in cell apoptosis. Based on these findings, we propose a model (Figure 6) describing dual damage to telomeres and mitochondria in T cells under oxidative stress. To the best of our knowledge, this is the first report showing dual injury and signaling crosstalk between telomeres and mitochondria in T cells under selective oxidative stress on a specific organelle.

Oxidative stress contributes to the pathogenesis of numerous diseases, including infectious and inflammatory diseases, cancer, cardiovascular disease, and neurodegeneration (Devasagayam et al., 2004; Hegde et al., 2012; Lonkar & Dedon, 2011; Malinin et al., 2011; Poljsak & Fink, 2014; Reuter et al., 2010). Previous studies have shown that oxidative stress accelerates telomere shortening and dysfunction through ROS‐induced base damage in telomeric DNA (von Zglinicki, 2002). We have recently shown telomere erosion and mitochondrial dysfunction in CD4^+^ T cells derived from virus‐infected individuals or from healthy subject CD4^+^ T cells treated with a telomere‐targeting drug (Schank et al., 2020; Zhao et al., 2021). By confining ^1^O_2_ formation to either telomeres or mitochondria in the present study, we demonstrated that human T cells exhibit accumulated telomeric DNA damage and compromised mitochondrial functions. Our results show that targeted oxidation‐induced telomere and mitochondrial injuries are time‐ and dose‐dependent, because the telomeric DNA damage and mitochondrial injury accumulated over time, leading to detrimental cellular apoptosis. Of note, the BER pathway has been shown to play a pivotal role in repairing DNA damage, especially for 8‐oxoG lesions (Fouquerel et al., 2019), as we have recently reported that PGC‐1α and mtTFA are repressed in CD4^+^ T cells and play a role in compromised mitochondrial functions in the setting of chronic viral infection (Schank et al., 2020; Zhao et al., 2021). In this study, however, we did not detect any changes in the expressions of PGC‐1α and mtTFA in T cells in the setting of targeted oxidative stress. Nevertheless, we discovered that selective oxidative stress at both telomeres and mitochondria remarkably suppressed XRCC1 expression, but not OGG1 and APE1, in stressed T cells. XRCC1 was also found to be suppressed in primary CD4^+^ T cells exposed to telomere‐targeting drug KML001 (Schank et al., 2020). Since XRCC1 exerts an essential role in DNA repair in the absence of ATP‐dependent DNA ligase III (LIG3) (Gao et al., 2011), our findings suggest that inhibition of XRCC1 in the BER pathway by oxidative stress might lead to failure of mtDNA damage repair, eventually resulting in mitochondrial dysfunction, telomere damage, and cell apoptosis. In addition, we showed evidence that multiple enzymes and/or signaling molecules are dysregulated in T cells undergoing oxidative stress (Figure 5), suggesting that these molecules may play a role in these oxidative stress‐caused cross‐injuries and cellular crisis. Specifically, COX17, TTC19, and CYCS are involved in mitochondrial respiration (Bottani et al., 2017; Punter et al., 2000; Zhao et al., 2003); RPA1 plays an essential role in both DNA replication and the cellular response to DNA damage (Lin et al., 1998); and AK2 has been implicated in cell apoptosis (Köhler et al., 1999). Further studies are warranted to investigate the mechanism by which these candidate proteins can induce telomere damage and mitochondrial dysfunction in the setting of oxidative stress.

A limitation of this study is that we used Jurkat cells to establish stable clones transfected with a telomere‐ or mitochondria‐targeting construct for our experiments. Jurkat cells are a human leukemia T cell line that is quite different from primary T cells in terms of their telomere length and mitochondrial metabolism. While primary T cells are more relevant in addressing oxidative injury at telomeres or mitochondria during inflammation and infection, we couldn't carry out these experiments using human primary T cells due to technical challenges. Nevertheless, in the present study we provide direct evidence that targeted oxidation on telomeres and mitochondria in Jurkat T cells causes dual injuries to these two important organelles and that these injuries accelerate and promote T cell senescence, dysfunction, and apoptosis. Interestingly, we observed a significant increase in MO, which reflects the level of mitochondrial membrane potential and oxidative phosphorylation, but a decrease in OCR (cellular respiration) during the chemoptogenic tool‐mediated oxidative stress. These results are in line with our previous findings in CD4^+^ T cells from latently HIV‐infected individuals on antiretroviral therapy (Zhao et al., 2021). However, in our studies in primary T cells treated with KML001 (a telomere‐targeting drug), which exhibit a high level of ROS, we observed an increase in MG (mitochondrial mass) but a decrease in MO and OCR (Schank et al., 2020). We believe that this discrepancy is due to the different experimental settings being used, for example, different cell types and treatments, which warrant further investigation. Also, we measured telomere length and mtDNA copy numbers in both cell lines exposed to light plus dye in the presence of NaCa. Although we observed decreases in ROS, MG, MO, and apoptosis, and increases in OCR, we did not find alterations in telomere length or mtDNA content, suggesting that NaCa only blocks ROS production and its downstream effects. These results are consistent with the mechanism of action of NaCa. Further studies are required to elucidate the molecular mechanism that can restore telomere length and mtDNA, which are critical for reversing cell aging in the setting of oxidative stress induced by infection and/or inflammation.

## 4 EXPERIMENTAL PROCEDURES

### 4.1 Cell lines, dye, and expression plasmids

Human Jurkat E6‐1 (E6‐1) and HIV‐1 LAV‐infected Jurkat E6 Cells (J1.1) were obtained from NIH AIDS Reagent Program, and cultured in complete RPMI 1640 media (10% FBS, 2 mM L‐glutamine, and 50 Unit/mL Penicillin‐Streptomycin). MG2I was provided by Dr. Marcel P. Bruchez (Carnegie Mellon University). The plasmid pCDNA3.1/COX IV‐COX VIII‐dL5‐2XG4S‐mCer3 was purchased from Addgene (Cat # 73208). In this plasmid, the dL5** (a Fluorogen Activating Protein (FAP)) is fused with mCerulean3 (mCer3) by 2 copies of a G4S linker. The mitochondrial targeting sequences of COX VIII and COX IV were tagged, which results in consistent mitochondrial localization of the fused protein FAP‐mCer3 (Telmer et al., 2015). The pLVX‐FAP‐mCer‐TRF1 plasmid was generously provided by Dr. Opresko (University of Pittsburg), and the fusion of TRF1 to FAP‐mCer3 leads to targeted telomere localization of the fused protein (Fouquerel et al., 2019).

### 4.2 E6‐1/mito‐FAP‐mCer3 and J1.1/FAP‐mCer3‐TRF1 stable cell lines

E6‐1 and J1.1 cells were transfected with pCDNA3.1/COX IV‐COX VIII‐dL5‐2XG4S‐mCer3 or pLVX‐FAP‐mCer‐TRF1, respectively, using the Amaxa Cell Line Nucleofector Kit V and program X‐001 (Lonza). After 48 h, the plasmid‐transfected and mock (dd H_2_O) transfected cells were selected in complete RPMI 1640 media containing 1 mg/mL of G418 for approximately 2 weeks, until all mock cells completely died. The mCer3^+^ (green) cells were sorted from the transfected cells with a BD FACSAria Fusion flow cytometer and cultured in complete RPMI 1640 media containing 1 mg/mL of G418.

### 4.3 Immunofluorescent staining and confocal microscopy

To investigate FAP‐mCer3‐TRF1 location on telomeres, the J1.1/FAP‐mCer3‐TRF1 cells were fixed in 2% PFA for 20 min, followed by permeabilization with 0.3% Triton X‐100 in PBS for 10 min. After blocking with 5% BSA in PBS for 1 h, the J1.1/FAP‐mCer3‐TRF1 cells were incubated with rabbit anti‐GFP antibody (Sigma‐Aldrich) and mouse anti‐TRF2 antibody (Novus Biologicals) at 4°C overnight. The cells were washed three times with PBS containing 0.1% Tween‐20, and then stained with anti‐rabbit IgG‐Alexa Fluor 488 and anti‐mouse IgG‐Alexa Fluor 555 (Thermo Scientific) at room temperature for 1 h, and then washed and mounted with DAPI Fluoromount‐G (SouthernBiotech). To investigate the mitochondrial localization of mito‐FAP‐mCer3, the E6‐1/mito‐FAP‐mCer3 cells were pre‐stained with MitoTracker Red CMXRos, and then stained with rabbit anti‐GFP antibody followed by incubation with anti‐rabbit IgG‐Alexa Fluor 488. To investigate DNA damages on telomeres, the J1.1/FAP‐mCer3‐TRF1 and E6‐1/mito‐FAP‐mCer3 cells were treated three times with MG2I and light exposure, stained with rabbit anti‐53BP1 (Cell Signaling) and mouse anti‐TRF2, incubated with respective secondary antibodies. Images were acquired with Leica DMi8 Confocal System (Leica Confocal).

### 4.4 Singlet oxygen induction

The protocol of singlet oxygen induction was conducted as previously described(Fouquerel et al., 2019; Qian et al., 2019). The E6‐1/mito‐FAP‐mCer3 and J1.1/FAP‐mCer3‐TRF1 cells were seeded onto 60 mm dishes. Each dish contained 6–8 million cells in 3.5 mL OptiMEM (Gibco) with or without 200 nM of MG2I (a photosensitizer dye di‐iodinated malachite green, generously provided by Dr. Marcel P. Bruchez from Carnegie Mellon University). The cells were then exposed to a 660 nm LED light at 24V/100W for 5, 10, or 20 min, to trigger excitation of the FAP‐bound MG2I dye and the production of singlet oxygen in mitochondria or at telomeres. Following the dye and light exposures, the cells were incubated for 30 min in a CO_2_ incubator at 37°C, then resuspended in complete RPMI 1640 medium and cultured for 24 h. The cells were treated with dye and/or light exposure every 24 h for 3–5 consecutive days.

### 4.5 Flow cytometry

To quantify cell apoptosis, Annexin V and 7‐AAD were measured using BD Pharmingen PE Annexin V Apoptosis Detection Kit I (BD Biosciences). MitoTracker Green FM (MG) and MitoTracker Orange CM‐H_2_TMRos (MO) (Invitrogen) were used to detect mitochondrial mass and oxidation, respectively. CellROX Green reagent (ThermoFisher) was used to measure cellular ROS. All the assays were performed according to the manufacturers' instructions. The stained cells were analyzed by an Accuri C6 flow cytometer (BD), and the data were analyzed by FlowJo software (Tree Star). Unstained cells were used to determine the background levels of staining and adjust multicolor compensation as a gating strategy.

### 4.6 Genomic DNA isolation and real‐time qPCR

Total genomic DNAs were isolated from the cells treated with dye and light exposure, using PureLink Genomic DNA Mini Kits (Invitrogen). Mitochondrial DNA (mtDNA) was detected using the following primers: forward 5′‐CACCCAAGAACAGGGTTTGT‐3′ and reverse 5′‐TGGCCATGGGTATGTTGTTA‐3′. Nuclear β2‐microglobulin DNA was detected using the following primers: forward 5′‐TGCTGTCTCCATGTTTGATGTATCT and reverse 5′‐TCTCTGCTCCCCACCTCTAAGT‐3′. SYBR Green real‐time qPCR reactions (Bio‐Rad) were run in triplicates under the following conditions: 1 cycle at 52°C for 5 min, 1 cycle at 95°C for 10 min, and 40 cycles at 95°C for 15 s and 62°C for 1 min (Venegas et al., 2011). Telomeric DNA (telDNA) was detected using the following primers (originally designed by Cawthon) (Cawthon, 2002): forward 5′‐ GGTTTTTGAGGGTGAGGGTGAGGGTGAGGGTGAGGGT‐3′ and reverse 5′‐ TCCCGACTATCCCTATCCCTATCCCTATCCCTATCCCTA‐3′. The 36B4 DNA was detected using the following primers: forward 5′‐CAGCAAGTGGGAAGGTGTAATCC‐3′ and reverse 5′‐ CCCATTCTATCATCAACGGGTACAA‐3′. The PCR conditions for telDNA were as follows: 1 cycle at 95°C for 10 min, and 31 cycles at 95°C for 15 s and 54°C for 2 min. The conditions for 36B4 DNA were: 1 cycle at 95°C for 10 min, and 30 cycles at 95°C for 15 s and 58°C for 1 min (Cawthon, 2002). The levels of mtDNA content and telomere length were calculated using the 2^−ΔΔct^ cycle threshold method and normalized, respectively, to β2‐microglobulin and 36B4 DNAs, and are expressed as fold change over the control samples.

### 4.7 Seahorse XFp cell mito stress assays

To measure the OCR (an indicator of mitochondrial respiration), the Cell Mito Stress Test was performed using a Seahorse XFp instrument according to the manufacturer's protocol (Seahorse, Agilent Technologies). One day prior to the assay, Seahorse mini cartridges were hydrated overnight in a non‐CO_2_ incubator. On the day of the assay, the E6‐1/mito‐FAP‐mCer3 and J1.1/FAP‐mCer3‐TRF1 cells treated with or without dye and light exposures were seeded onto mini culture plates, which were precoated with poly‐D‐lysine (ThermoFisher Scientific). Approximately, 100,000 cells per well were cultured in Seahorse XF RPMI assay medium supplemented with 1.0 mM of glucose, 100 µM of pyruvate, and 1.0 mM of glutamine. The following inhibitors from the Cell Mito Stress Test kit were added to the culture media in this order: 2.0 μM of oligomycin, 1.5 μM of FCCP, and 2.0 μM of rotenone/antimycin A, and the related three sequential measurements were taken. Data analysis was performed using the Seahorse Wave software and the Seahorse mito stress test report generator.

### 4.8 N‐acetylcysteine amide (NaCa) treatment

N‐acetylcysteine amide (a ROS inhibitor) was purchased from Sigma‐Aldrich. The E6‐1/mito‐FAP‐mCer3 and J1.1/FAP‐mCer3‐TRF1 cells were pre‐treated with DMSO or 1 mM of NaCa for 30 min at 37°C, followed by dye and light (20 min) exposures. After recovery for 1 h in a 37°C incubator, NaCa was removed by washing the cells with fresh complete RPMI 1640 medium, and the cells were cultured for 24 h. Then, the treatments were repeated every 24 h for 3 or 5 consecutive days and harvested for measuring cell apoptosis, MG, MO, cellular ROS, and mitochondrial respiration.

### 4.9 Western blots

The cells were lysed on ice in RIPA lysis buffer (Boston BioProducts) in the presence of a protease inhibitor cocktail (Sigma‐Aldrich). The protein concentrations were measured by Coomassie blue stains (Bio‐Rad). The proteins were separated by SDS‐PAGE, transferred to nitrocellulose membranes, which were blocked with 5% non‐fat milk, 0.1% Tween‐20 in Tris‐buffered saline, and incubated with anti‐GFP antibody (Sigma‐Aldrich), or anti‐OGG1, anti‐XRCC1, anti‐APE1, anti‐PGC‐1α, anti‐NRF‐1, anti‐ERRα, or anti‐mtTFA antibodies (Cell Signaling). After incubation with appropriate horseradish peroxide (HRP)‐conjugated secondary antibodies (Cell Signaling), the proteins were detected using the Amersham ECL Prime Western Blotting Detection Reagent (GE Healthcare BioSciences). Protein bands were captured and quantified using the ChemiDoc MP Imaging System (Bio‐Rad).

### 4.10 Liquid chromatography‐tandem mass spectrometry (LC‐MS)

Active and intact mitochondria from the treated E6‐1/mito‐FAP‐mCer3, J1.1/FAP‐mCer3‐TRF1, and control cells were purified using the Qproteome Mitochondria Isolation kit (Qiagen). The mitochondrial proteins were assessed using an LC‐MS shotgun approach at the Mass Spectrometry Facility of Rutgers University. Briefly, after resolving the protein lysates onto 1.5 mm thick 10% NUPAGE Bis‐Tris gel (Thermo Fisher) as a gel plug, the gel was stained with Coomassie R250 and destained using standard procedures. The gel bands were excised for in‐gel digestion using sequencing‐grade trypsin and then analyzed by LC‐MS L using Nano LC‐MS/MS (Dionex Ultimate 3000 RLSCnano System) interfaced with QExactive HF. Label‐free quantification (LFQ) was used to determine the relative amount of proteins as fold changes, calculated based on the power 2 of LFQ differences of the dye plus light‐treated group and the control group. Since there are 3 control groups (untreated, only dye, and only light), to simplify the data analysis, the representative LFQ of the control group was the average of LFQs of the three control groups. The graph of the LC‐MS data set was generated using Prism 7 software.

### 4.11 Statistical analysis

Data were analyzed using Prism 7 software and are expressed as mean ± SEM. For Figures 1c–f, 2d–j, 3c–g, 4a,b, 5b,d, Figure S1a–e, comparisons among multiple groups were made using a one‐way ANOVA at a 95% confidence level (Tukey's honest significance test). For Figure 4c–l, Figure S1f,g, comparisons between two groups were made using a parametric paired *t*‐test for normally distributed data or non‐parametric Wilcoxon paired *t*‐test for non‐normal distributions. *p*‐values of <0.05 (*), <0.01 (**), <0.001 (***) or <0.0001 (****) were considered statistically significant or very significant, respectively.

## CONFLICT OF INTEREST

None declared.

## AUTHOR CONTRIBUTIONS

L.W. and Z.Y.L. performed most of the experiments, J.Z., M.S., D.C., X. D., L.N.N., L.N.T.N., and S. K. performed some experiments. J.P.M. coordinated human subject recruitment. X.Y.W provided technical support. M.E., J.Y.Z., S.N., and J.P.M. offered intellectual input for troubleshooting and discussion of the findings. Z.Q.Y. supervised the research and wrote the manuscript, with the help of all other co‐authors.

## Supporting information

Fig S1‐S2

## Data Availability

The datasets generated and analyzed during the course of this study are available from the corresponding author upon reasonable request. The data sharing policies will be followed per NIH and VA guidelines.
