# Supplementary figures and images for: Selective oxidative stress induces dual damage to telomeres and mitochondria in human T cells
Source: Aging Cell. 2021 Nov 9;20(12):e13513. doi: 10.1111/acel.13513 (PMC8672791; doi:10.1111/acel.13513)

**Fig.S1**

**a**

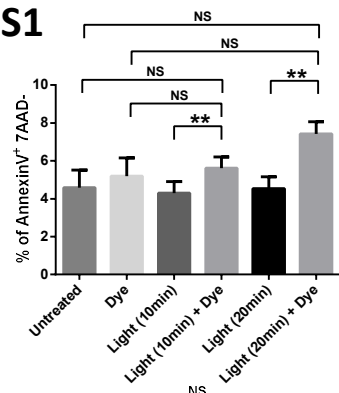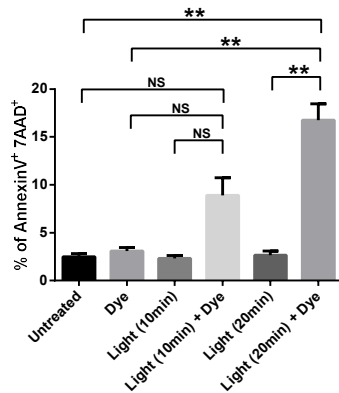

**b**

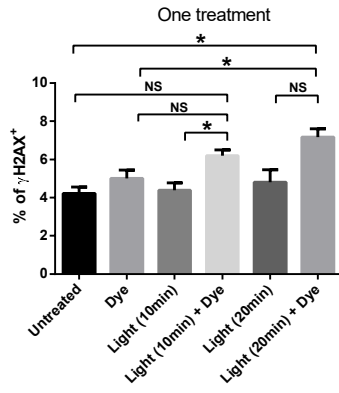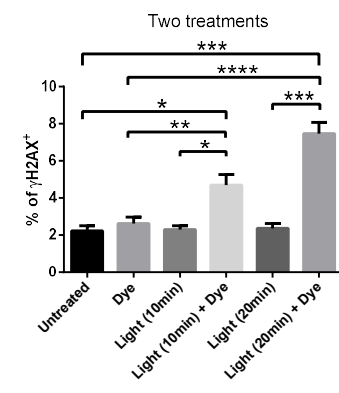

**c**

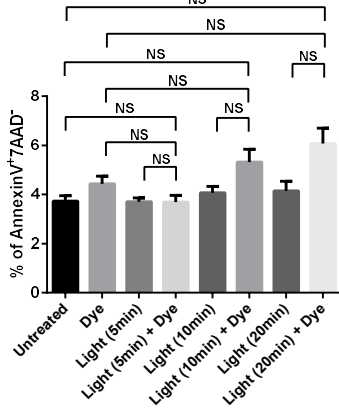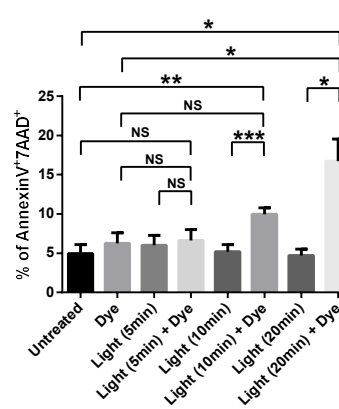

**d**

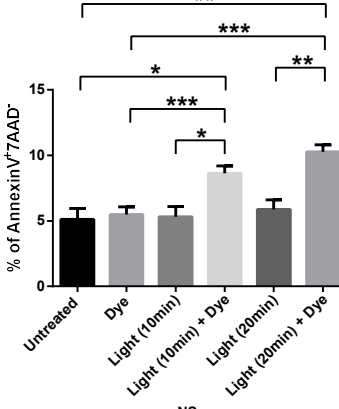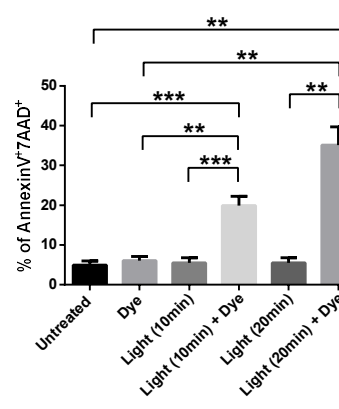

**e**

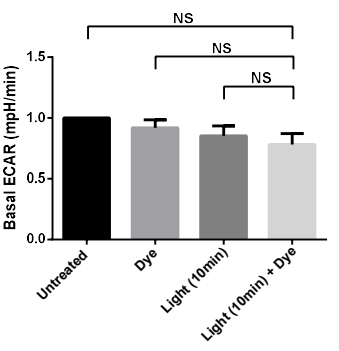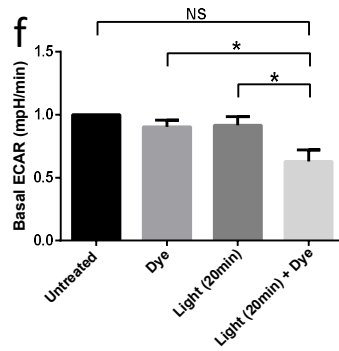

**g**

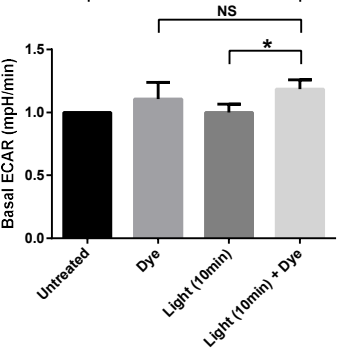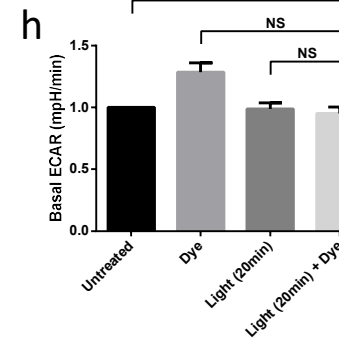

Fig.S2

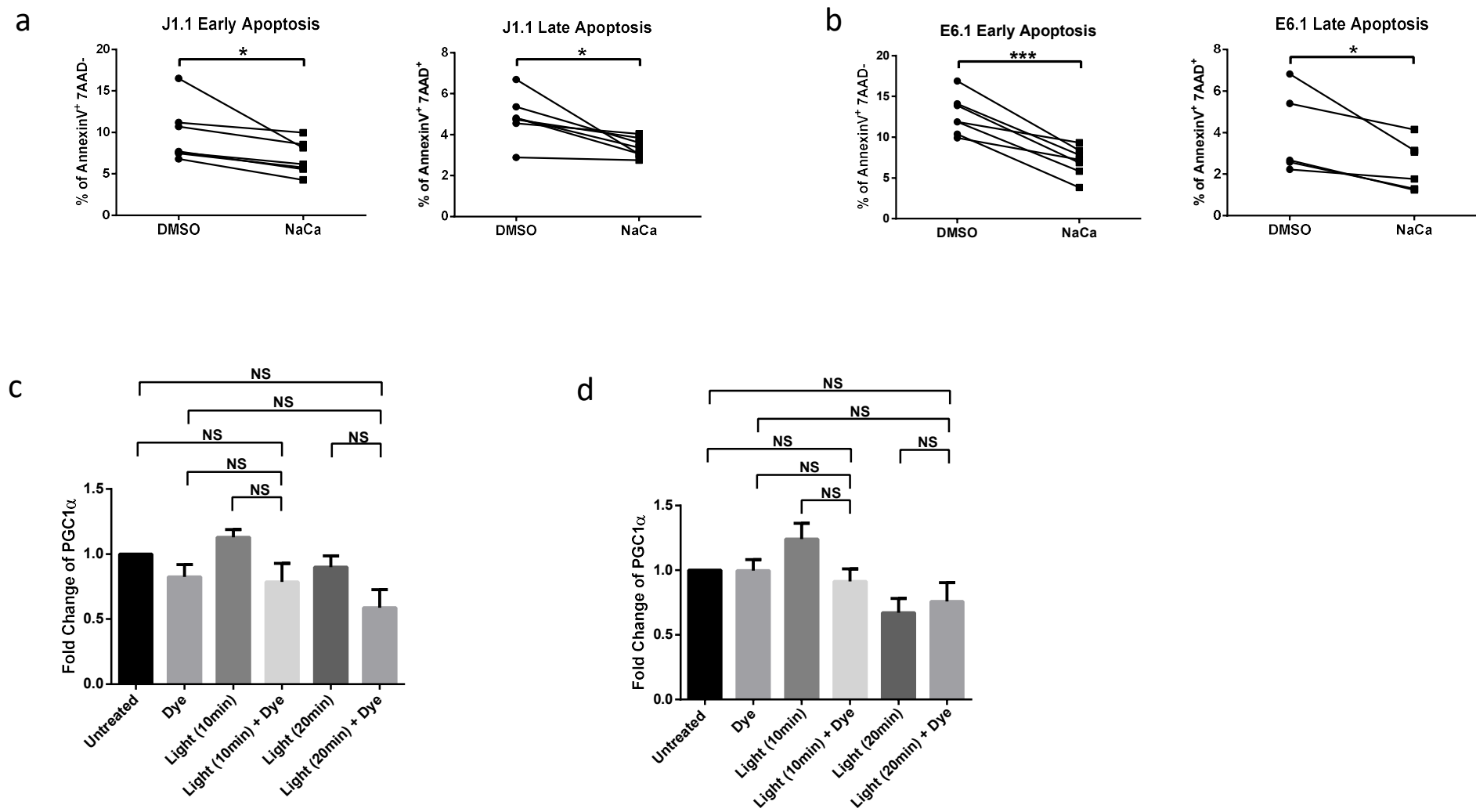

Supplement: Supplementary file 1 — Fig S1‐S2 [file ACEL-20-e13513-s001.pdf]
